# Pulsed Electromagnetic Fields (PEMFs) Trigger Cell Death and Senescence in Cancer Cells

**DOI:** 10.3390/ijms25052473

**Published:** 2024-02-20

**Authors:** Pavlos Pantelis, Giorgos Theocharous, Dimitris Veroutis, Ioanna-Aglaia Vagena, Aikaterini Polyzou, Dimitris-Foivos Thanos, Efthymios Kyrodimos, Athanassios Kotsinas, Konstantinos Evangelou, Nefeli Lagopati, Vassilis G. Gorgoulis, Nicholas Kotopoulos

**Affiliations:** 1 Molecular Carcinogenesis Group, Department of Histology and Embryology, Medical School, National and Kapodistrian University of Athens (NKUA), 11527 Athens, Greece; 2 Laboratory of Biology, Department of Basic Medical Sciences, Medical School, National and Kapodistrian University of Athens (NKUA), 11527 Athens, Greece; 3 1st ENT Department, Hippocration Hospital, National and Kapodistrian University of Athens (NKUA), 11527 Athens, Greece; 4 Biomedical Research Foundation, Academy of Athens, 11527 Athens, Greece; 5 Ninewells Hospital and Medical School, University of Dundee, Dundee DD1 9SY, UK; 6 Faculty Institute for Cancer Sciences, Manchester Academic Health Sciences Centre, University of Manchester, Manchester M20 4GJ, UK; 7 Faculty of Health and Medical Sciences, University of Surrey, Surrey GU2 7YH, UK

**Keywords:** pulsed electromagnetic fields (PEMFs), cancer therapeutics, cell viability, cell death, senescence

## Abstract

The currently available anti-cancer therapies, such as gamma-radiation and chemotherapeutic agents, induce cell death and cellular senescence not only in cancer cells but also in the adjacent normal tissue. New anti-tumor approaches focus on limiting the side effects on normal cells. In this frame, the potential anti-tumor properties of Pulsed Electromagnetic Fields (PEMFs) through the irradiation of breast cancer epithelial cells (MCF-7 and MDA-MB-231) and normal fibroblasts (FF95) were investigated. PEMFs had a frequency of 8 Hz, full-square wave type and magnetic flux density of 0.011 T and were applied twice daily for 5 days. The data collected showcase that PEMF application decreases the proliferation rate and viability of breast cancer cells while having the opposite effect on normal fibroblasts. Moreover, PEMF irradiation induces cell death and cellular senescence only in breast cancer cells without any effect in the non-cancerous cells. These findings suggest PEMF irradiation as a novel, non-invasive anti-cancer strategy that, when combined with senolytic drugs, may eliminate both cancer and the remaining senescent cells, while simultaneously avoiding the side effects of the current treatments.

## 1. Introduction

Cancer is one of the most prevalent causes of death worldwide. This entity represents a multifaceted disorder commonly characterized by heterogeneity [1]. Thus, complex treatments are required, while the continuous development of novel approaches that either eliminate or delay the growth of cancer cells comprises an imperative therapeutic goal [2].

Chemotherapy, radiation therapy [3], radionuclide therapy [4], immunotherapy and hormone therapies along with surgery are traditionally applied, solely or in combination, and are considered as powerful tools in our arsenal against cancer. As far as radiation therapy is concerned, ionizing irradiation [characterized by very low wavelength, high frequency and low linear energy transmission (LET)] [5] is conventionally utilized as the treatment of choice, resulting in the shrinkage or remission of tumors [6]. However, following this intervention, serious side effects usually occur at the local and systematic level, e.g., secondary cancer, Radiation-Induced Coronary Artery Disease (RICAD) [7], etc. Locally, adjacent tissues are also affected, given that this type of irradiation is harmful not exclusively to cancer cells but also to normal ones. Furthermore, radiation therapy is not appropriate for all patients, due to several endogenous vulnerabilities in some categories of patients [8].

Compared to normal cells, cancer cells harbor a variety of structural abnormalities [9,10]. Such defects are associated with alterations in the genome (genomic instability) and a plethora of altered domains/organelles such as the nuclear envelope, the cytoskeleton, mitochondria, centrosomes, and the cell membrane. Collectively, they reflect the architectural “anarchy” that cancer cells exhibit, rendering them susceptible to mild interventions that would putatively not harm normal cells [9,10,11,12,13,14,15]. Various studies have previously focused on the use of Photodynamic Therapy (PDT) [16] and Photothermal Therapy (PTT) [17] in parallel with conventional treatments. They rely on applying Infrared (IR) or Ultraviolet (UV) light in order to target cancer cells, avoiding any harmful effect(s) on normal ones. In this context, we hypothesized whether low-intensity radiation could be applied for effective cancer therapeutic purposes, exploiting the specific characteristics of cancer cells, while at the same time leaving normal cells and tissues adjacent to the tumor unaffected.

Pulsed Electromagnetic Fields (PEMFs) represent a type of low-intensity irradiation. According to the general categorization of these electromagnetic frequencies by the IEEE (Institute of Electrical and Electronics Engineers), ULFs are Ultra Low electromagnetic Frequencies (<3 Hz), ELFs are Extremely Low electromagnetic Frequencies (3 Hz–30 kHz) and VLFs are Very Low electromagnetic Frequencies (30–300 kHz) [18]. In the past few years, PEMFs have been a point of interest for the medical community, since they appear to have a wide range of applications. Importantly, they have been used as a supporting therapy, improving the healing rate and accelerating the recovery period in post-surgery patients [19], since PEMFs facilitate biophysical interactions. It is suggested that PEMFs might be beneficial at the cellular/organismal level, such as in the recharging of the Trans-Membrane Potential (TMP), the increase in adenosine triphosphate (ATP) production in the mitochondria, the enhancement of the sodium–potassium pump, the increase in cellular pH, the improvement of oxygen uptake and its assimilation into cells, the reduction in blood viscosity and improved circulation [20]. In this context, radiation of low energy and frequency may have/provoke various beneficial effects in treating different pathologies. Focusing on ELF-PEMFs, as a pathology treatment approach, they have mainly been used as a complementary type of therapy, coupled with chemo-/radiotherapy, etc., and rarely on their own, as a monotherapy. There are sparse indications in the literature regarding their putative promising role in cancer treatment [21]. Consequently, considering the fragile profile of cancer cells and the beneficial potential of PEMFs acting as an anticancer agent, traditional radiation therapy might also incorporate PEMF treatment sessions in order to decrease the resistance of cancer cells to other therapies, strengthening the killing effect on such cells, while also nullifying potential side effects on normal cells. In the current work, we examined the aforementioned working hypothesis by applying ELF-PEMFs on breast cancer epithelial cells as well as normal fibroblasts.

## 2. Results and Discussion

### 2.1. Experimental Design

**Figure 1:**
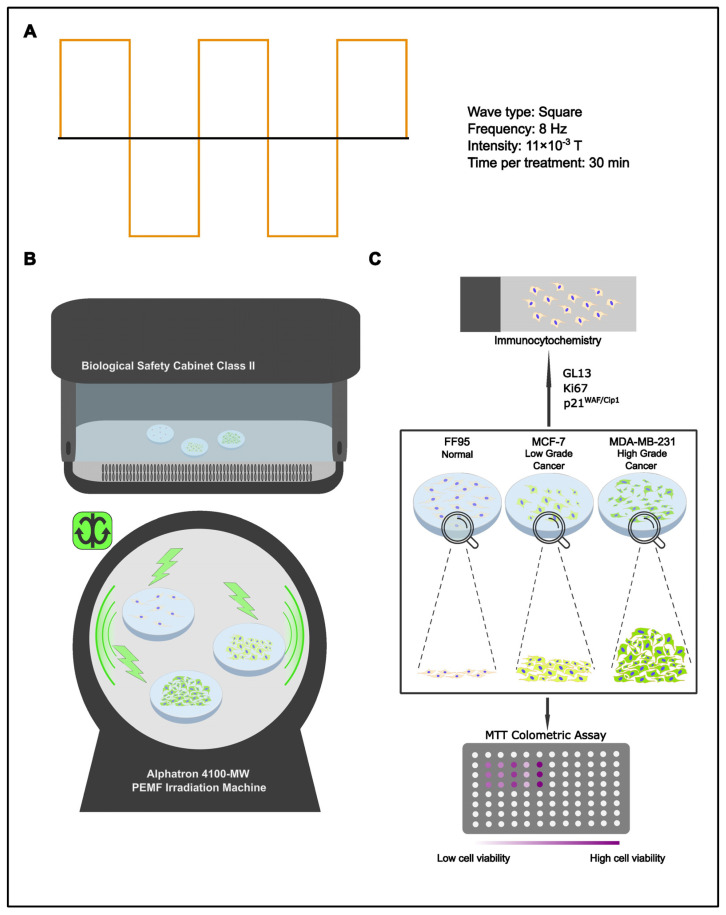
Experimental design. (**A**) Settings of applied manual program 3 for cell treatment. (**B**) Schematic representation of PEMF treatment. Cells were irradiated with PEMFs or remained in the laminar flow cabinet (control group). (**C**) Treated cells and corresponding controls were evaluated for cell viability, proliferation rate and cellular senescence employing the MTT assay, Trypan blue staining and immunocytochemical staining for Ki67, p21^WAF1/Cip1^ and GL13, respectively.

Tumor complexity restricts therapeutics to a handful of generalized, non-invasive approaches. Chemo-/radiotherapy are two of the most established strategies in the anti-cancer arsenal; they are, however, far from flawless. Their mercurial nature, although effective against cancer cells, has proven equally harmful towards the normal surrounding tissue. In spite of cancer’s unpredictable behavior/outcome, what the pathology gains in numbers through enhanced proliferating rates it lacks in stability and cellular component integrity. This key characteristic could be utilized in order to identify the break-point at which a therapeutic approach could be detrimental against cancer but at the same time harmless towards non- pathological cells. Since high-energy/short-wavelength radiation (radiotherapy) has deleterious effects on normal cells, the effect of low-energy/high-wavelength radiation may provide an alternative, less harmful treatment option. Hence, various pre-set programs of the Alphatron machine were put to the test. Data collected from these experiments led to the development of a manual PEMF irradiation program that consists of a frequency of 8 Hz, a wave type of full square, magnetic flux density of 0.011 T and a duration of 30 min two times per day for 5 consecutive days (Table 1) (Figure 1A). Since breast cancer is the most common cancer type in women overall [22], the corresponding low-grade (MCF-7) and high-grade (MDA-MB-231) cell lines, along with normal fibroblast cells (FF95), were selected in the current study. For each cellular type, the same groups of cells were PEMF-irradiated or remained in the laminar flow hood as the non-treated control (Figure 1B). With the aforementioned aim in mind, the experiment was conceptualized to investigate the in vitro effects of PEMFs on cell viability, proliferation rate and the induction of cellular senescence in breast cancer cells and normal fibroblasts (Figure 1C).

### 2.2. Pulse Electromagnetic Fields (PEMFs) Trigger Cell Death and Cellular Senescence

#### Effect on Cell Proliferation Rate

**Figure 2:**
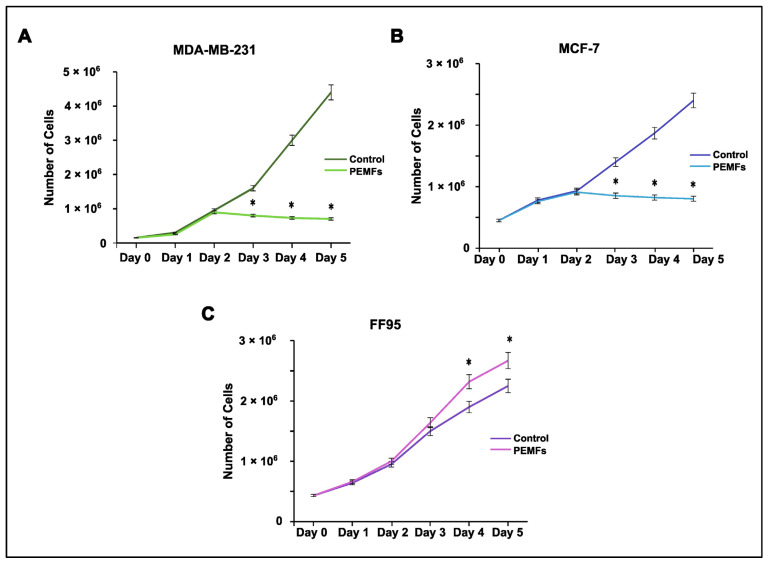
Effect of PEMF irradiation on cell proliferation. Trypan blue staining and cell counting was performed to estimate the proliferation rate of PEMF-treated and control cells. (**A**) MDA-MB-231 and (**B**) MCF-7 cells exhibited decreased proliferation capacity following PEMF application in comparison to untreated cells. (**C**) PEMF irradiation in FF95 cells was accompanied by a statistically significant increase in proliferation in contrast to unirradiated controls. Statistical analysis was performed through the Kruskal–Wallis nonparametric test. The obtained data represent means ± standard deviation from three experiments. * *p* < 0.05.

In order to investigate the potential effects of PEMF irradiation as an anti-tumor strategy, we estimated the proliferation rate of breast cancer cells as well as normal fibroblasts. Cells were stained with Trypan Blue and counted following PEMF treatment or in the absence of it. MCF-7 and MDA-MB-231 cells displayed a statistically significant decrease in their proliferation capacity compared to the controls (Figure 2A,B). Interestingly, the irradiated FF95 cells showed an increased proliferation rate (Figure 2C). Collectively, these data indicate that PEMF irradiation exhibited not only anti-cancer properties but also beneficial effects for the normal cells.

### 2.3. Effect on Cytotoxicity

**Figure 3:**
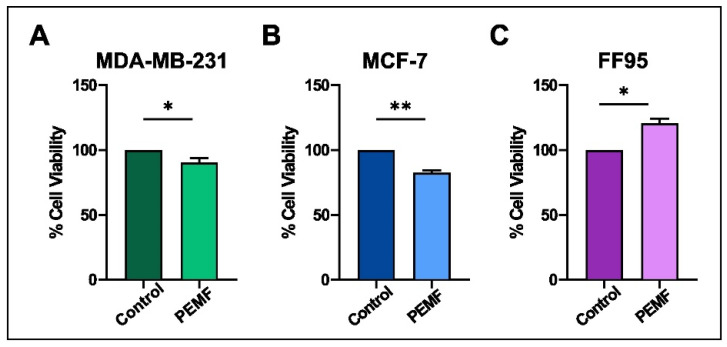
Evaluation of cytotoxic effect after PEMF treatment in breast cancer cells and normal fibroblasts. MTT colorimetric assay was employed to estimate the percentage of cell viability of MDA-MB-231, MCF-7 and FF95 cells in the presence or absence of PEMF irradiation. PEMF treatment gradually decreased the cell viability of (**A**) MDA-MB-231 and (**B**) MCF-7 compared to the corresponding controls, while this phenomenon was opposite in FF95 cells (**C**). Statistical analysis was performed through the Wilcoxon nonparametric test. The obtained data represent means ± standard deviation from four experiments. * *p* < 0.05, ** *p* < 0.005.

Cell viability was measured by the MTT colorimetric assay in all PEMF-treated and control cellular systems. Notably, we found decreased levels in the viability of breast cancer cells; particularly, 10% and 16% reductions were observed in MDA-MB-231 and MCF-7, respectively (Figure 3A,B). Interestingly, the corresponding results in FF95 cells indicated not only a non-cytotoxic effect in cell viability but also a 20% significant increase in it (Figure 3C). The latter finding in combination with the decreased proliferation rate of FF95 cells revealed another aspect of PEMFs that could be advantageous in the field of surgery regarding tissue repair and wound healing [23]. Particularly, the observed increased cell viability of fibroblasts induced by PEMFs could prove useful for post-operation due to the boost in their regenerative capacity.

### 2.4. Detection of Cellular Senescence

Subsequently, we evaluated the proliferation rate and cellular senescence of PEMF-irradiated cells compared to unirradiated ones. Specifically, data on proliferation rates were obtained by estimating Ki67 immunostaining, cellular senescence through the presence of lipofuscin (GL13 staining) and the immunohistochemical expression of p21^WAF1/Cip1^. Cellular senescence is a stress response mechanism characterized by a state of generally irreversible cell cycle arrest. Apart from the absence of proliferation, senescent cells exhibit distinct morphological and molecular characteristics, including macromolecular damage, disrupted metabolism and the secretion of a unique set of molecules collectively known as the senescence-associated secretory phenotype (SASP) [24]. Cellular senescence plays a crucial role in preventing the proliferation of stressed, damaged and potentially harmful cells, such as those with extensive DNA damage. While senescence contributes to maintaining tissue homeostasis and suppressing the development of cancer, it also plays important roles in organismal development, aging and age-related diseases. The accumulation of senescent cells over time contributes to tissue dysfunction and various age-related pathologies [24,25]. Lipofuscin is a pigment of oxidized proteins, lipids and metals that accumulates within the lysosomes and cytoplasm of senescent cells. Lipofuscin is considered as a hallmark of senescence and is recognized by the GL13 (SenTraGor^TM^) reagent [26]. Importantly, GL13 staining is the first step of a proposed algorithm from the senescence community for the accurate detection of senescent cells. The second step includes the co-detection of other markers related to cell cycle arrest such as p16^INK4A^ and p21^WAF/Cip1^ or the absence of proliferation markers, such as Ki67 [26].

**Figure 4:**
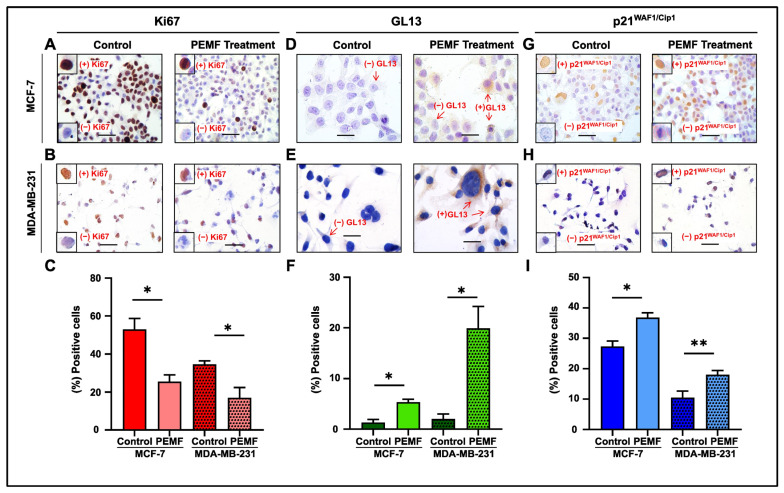
Evaluation of proliferation and cellular senescence in breast cancer cells following PEMF treatment. Representative images of Ki67 (**A**,**B**), GL13 (**D**,**E**) and p21^WAF1/Cip1^ (**G**,**H**) immunocytochemical staining. Positive cells were calculated by evaluating the strong brown nuclear signal for Ki67 and p21^WAF1/Cip1^. GL13 staining exhibited a strong brown perinuclear and/or cytoplasmic signal. Graphs depict the percentage of positive cells (%) for Ki67 (**C**), GL13 (**F**) and p21^WAF1/Cip1^ (**I**) in PEMF-treated and control cells. Approximately 100 cells per optical field were counted, and ≥5 high-power fields per sample were used for the quantification. Statistical analysis was performed employing the Wilcoxon nonparametric test. The data obtained represent means ± standard deviation from four independent experiments. * *p* < 0.05, ** *p* < 0.005. Objective 20×, 40×. Scale bars: (**A**,**B**,**G**,**H**) 30 μm, (**D**,**E**) 60 μm.

In this report, PEMF application demonstrated a decrease in proliferation, as indicated by the reduced Ki67 expression, in MCF-7 and MDA-MB-231 cells compared to controls (Figure 4A,B). Quantification analysis revealed a statistically significant equal decrease in Ki67 in both cancer cell lines (Figure 4C). Moreover, this treatment increased senescence, as assessed by GL13 and p21^WAF/Cip1^ staining in MCF-7 and MDA-MB-231 cells compared to the corresponding untreated cells (Figure 4D–I). Results showed that this statistically increased levels of cellular senescence following PEMF irradiation was only in the irradiated cells. In accordance with the previously proposed algorithm, GL13 overlapped with p21^WAF1/Cip1^ staining and exhibited a mutually exclusive pattern with Ki67 [26].

**Figure 5:**
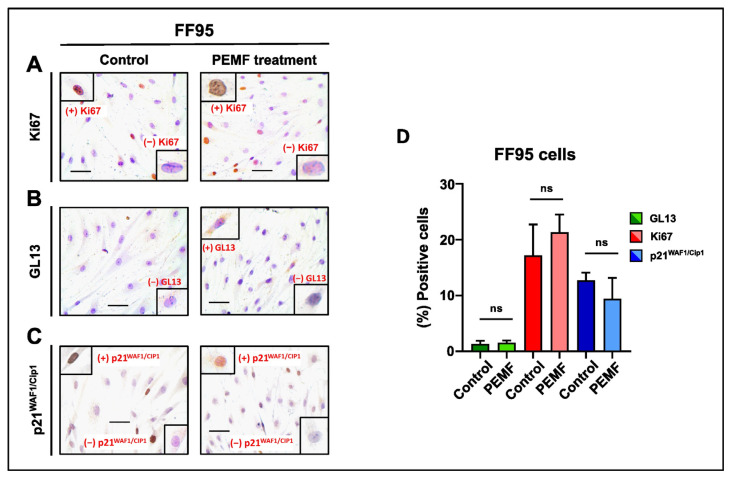
Evaluation of proliferation and cellular senescence in normal fibroblasts (FF95) following PEMF treatment. Representative images of Ki67 (**A**), GL13 (**B**) and p21^WAF1/Cip1^ (**C**) immunocytochemical staining. Ki67 and p21^WAF1/Cip1^ positivity was determined by evaluating the strong brown nuclear signal. GL13 staining exhibited a strong brown perinuclear and/or cytoplasmic signal. (**D**) Graph depicting the percentage of positive cells (%) for Ki67, GL13 and p21^WAF1/Cip1^ in PEMF-treated and control cells. Approximately 100 cells per optical field were counted, and ≥5 high-power fields per sample were used for the quantification. Statistical analysis was performed employing the Wilcoxon nonparametric test. The obtained data represent means ± standard deviation from four experiments. ns: non-significant. Objective 20×. Scale bar: 30 μm.

Notably, no significant change in the proliferation rate and cellular senescence was observed in the FF95 cells (Figure 5A–D). This comparative study shows that the exposure of different cells to a low intensity and frequency (8 Hz) of electromagnetic field, which is the exact frequency of the Earth’s magnetic field, could have a potential therapeutic window between normal and cancer cells. Several investigations have demonstrated that symmetrical, low-intensity electromagnetic fields (PEMFs) can inhibit cancer cells’ proliferation [21,27,28]. Our findings are in accordance with a previous study that used the low-intensity, frequency-modulated (6–25 Hz) Thomas electromagnetic field (EMF) pattern [21]. The Thomas EMF was able to inhibit the growth of cancer cell lines including B16-BL6, MCF-7, MDA-MB-231 and HeLa via increased Ca^2+^ uptake through T-type Ca^2+^ channels but did not affect the growth of normal cells. Additionally, Crocetti and colleagues showed similar results in an in vitro PEMF treatment study using cancer and non-cancer breast cell lines [29]. Importantly, the novelty of this study is that apart from the decreased proliferation rate as well as the reduction in cell viability, we revealed for the first time the induction of senescence in breast cancer PEMF-treated cells [21].

The multiple ways that PEMFs exert their effects include the transmission of electric signals on the different ions of the cells, the activation of specific pathways, the effect on the expression of various molecules, the compromise of the plasma membrane integrity, the changes in the mitochondrial pathways of energy and the inhibition or the activation of specific cell receptors [28,30,31,32]. Numerous theories including variations in temperature, flux density, or energy input have been examined to explain these changes in cellular responses [5]. The Alphatron 4100-MW (JCD Technology GmbH, Hamburg, Germany), used in our experimental procedure is a complex time-, frequency-, intensity- and wave-type-modulated instrument which could change during exposure. We propose that the aforementioned pattern (8 Hz, full square, magnetic flux density of 0.011 T) is critical for its effects on cells. Since the energy profiles of these exposures are equal, the effect of the PEMFs was not provoked by an augmentation in energy exposure and was more consistent with models of stochastic resonance.

Recent scientific reports support the beneficial effects of PEMFs-EMFs in vitro [33]. These effects are directly intertwined with the settings of the applied treatment for each case. Specifically, the beneficial biological effects that have been reported are partially ascribed not only to the amplitude, frequency, modulation, intensity, wave type and duration of exposure of the applied PEMF-EMF program, but also to the biological systems [34,35]. However, the biological mechanisms that have been proposed to explain the action of PEMFs-EMFs in biological systems are restricted [36,37]. In the work of Ayşe et al., an ELF-PEMF program of 50 Hz and 100 G exposure [38] induced the apoptosis and necrosis of K562 and U937 cells; noteworthily, a proliferation decrease (15% approximately) was observed in HeLa and PC-12 cells after 72 h of exposure to ELF-PEMF settings of 60 Hz and 1.2 ± 0.4 mT [39]. Moreover, a different time-varying application of ELFs, with dissimilar amplitudes but at the identical frequency (at 50 Hz) with the aforementioned work on PC-12 cells, resulted in proliferation reduction, as well as the induction of morphological differentiation [40]. In addition, in a recent publication [41], 24 and 72 h of exposure of MDA-MB-231 cells and SW-480, HCT-116 colon cancer cells to PEMF therapy (50 Hz; 10 mT) resulted in increased apoptosis in the MDA-MB-231 (55% and 20%), SW480 (11% and 6%) and HCT-116 cell lines (2% and 3%), respectively, compared with untreated control cancer cell lines. As a consequence, the different settings of the PEMF-EMF programs can result in discrete advantageous biological effects [42]. Further investigation can provide new insight on how to approach this phenomenon.

Evidently, there is a clear window of vulnerability of cancer cells following PEMF application. The validity of the described window effect is implicitly substantiated within the context of our data presented herein, not only due to the fact that the measuring of cell viability gave identical results, but also because there was a reduction in the proliferation rate of all the malignant cell lines. Though they have been documented and widely debated in the literature, similar window phenomena in electromagnetics have not been satisfactorily explained by any established model [29]. The interpretation of several observed biological effects of AM (amplitude modulated) electromagnetic fields is further complicated by the apparent existence of a window of response in both power density and the frequency domains, according to the Protection Guidelines Report of the International Commission on Non-Ionizing Radiation. No established theories can fully account for this occurrence, and that is the reason why this scientific work is a first step to approach this phenomenon. Nevertheless, more investigations are required in the future to explore the biochemical and molecular events that take place in cells following PEMF irradiation [43].

The cardinal outcomes of the current study include the non-cytotoxic effect of PEMFs in non-cancer cells, in contrast to the high levels of cellular death and senescence exhibited in PEMF-treated cancer cells. Both processes are considered as anti-tumor barriers, as cellular senescence has been recently characterized as a hallmark of cancer [44,45]. The ultimate goal of the current anti-cancer strategies is the elimination of the cell proliferation of cancer cells or the delay of the carcinogenesis progress. In this context, Therapy-Induced Senescence (TIS) is among the main scopes of the standard anti-cancer treatments and can be activated by different chemicals, chemotherapeutic drugs or gamma-irradiation [46]. However, these approaches lead to a plethora of side effects on adjacent normal cells [46,47]. According to our findings, PEMFs could be a useful tool to precondition the cancer tissue by inducing senescence in cancer cells without disrupting the homeostasis of non-cancer ones. The last stage of TIS includes the treatment of senescent cells with senolytic drugs that interfere with the anti-apoptotic pathways of senescent cells, allowing them to undergo apoptosis [48]. Our team has revealed the crucial role of senescent cells in various pathological entities including cancer, marking them as therapeutic targets [49]. Despite cellular senescence being an anti-tumor barrier, senescent cells exhibit a dual nature, as they could serve as a source for the progression to malignancy either through the expression of SASP molecules or due to their ability to escape from senescence. SASP molecules contribute to neo-angiogenesis, promote metastases by inducing changes in the tissue microenvironment via the production of metalloproteinases (MMPs) and create an immunosuppressive tissue microenvironment supportive for cancer progression in vivo and in vitro [48]. Senescent cells are associated with a generally permanent cell cycle arrest, but under certain conditions, re-entry in a proliferative status can occur. This phenomenon is termed escape from senescence [50]. Apart from the SASP, escaped cells have been associated with tumor relapse because they acquire highly aggressive features. In this context, the application of senolytic drugs at the proper time not only can selectively remove them, thus preventing the phenomenon of escape, but also reduce the SASP factors, avoiding their harmful effects. In order to achieve that, we need to have in our arsenal reliable tools for the detection and visualization of senescent cells. Recently, our team created a new reagent termed GLF16 that enables the in vitro and in vivo detection, isolation and live tracking of senescent cells [50]. GLF16, embedded in a nanocarrier (m-GLF16), permits the detection of living senescent cells. The linking of m-GLF16 with senolytic drugs provides a potentially innovative and appealing strategy in the field of theranostics, as it will reduce the side effects of the traditional therapeutic approaches. Collectively, PEMF treatment in cancer cells/tissues followed by the application of senolytics, ideally with tools such as m-GLF16, could be a novel, prominent and efficient non-invasive strategy for tumor elimination.

## 3. Materials and Methods

### 3.1. Cell Cultures

FF95 normal dermal fibroblasts, MDA-MB-231 and MCF-7 breast cancer cell lines, purchased from ATCC (MDA-MB-231: HTB-26^TM^, MCF7: HTB-22^TM^) (LGC Standards GmbH, ATCC, Wesel, Germany), were used for the in vitro experiments in this study. MDA-MB-231 is a human breast cancer cell line that does not express estrogen, progesterone and glucocorticoid receptors, while MCF-7 is less aggressive and invasive than the MDA-MB-231 breast cancer line. Cells were cultured in Dulbecco’s modified Eagle’s high glucose medium (DMEM) (Gibco BRL, Life Technologies, ThermoScientific, Paisley, UK), supplemented with 10% FBS and antibiotics (1% penicillin/streptomycin) (Gibco BRL, Life Technologies, Thermo Scientific, Paisley, UK), at 37 °C, 5% CO_2_ and were grown up to 70% confluence. The passage number of all the aforementioned cell lines was approximately 12–15. The cells were split twice a week, and after seeding, they were incubated for 24 h [51]. Subsequently, they were exposed to different conditions of PEMFs (for mention of the device/model, etc., see Section 3.2).

### 3.2. In Vitro PEMF Irradiation Device

#### Multi Pulse Magnetic Field Therapy Device, Alphatron 4100-MW

Cell treatments with PEMFs were performed using an Alphatron, JCD Technology GmbH (Hamburg, Germany), instrument that offers eight different preset programs as well as the ability to create custom ones through various different parameters. More precisely, the device allows one to manually adjust the desired treatment by selecting the suitable pulse wave form, desired frequency, treatment time, and the intensity of the treatment. This device was first programmed with the preset program C4 in order to test the effect of PEMFs and other manual programs (Table 1). These programs were applied to the tested cells twice per day (8 h gap between treatments) for 30 min. After applying these programs in all cell lines, a manual program with the following parameters was investigated: a frequency of 8 Hz, a wave type of full square, a treatment time of 30 min (2 times per day, for 5 days) and a magnetic flux density of 0.011 T. These settings were used for all subsequent experiments. The medium in the cell culture was removed and fresh medium was added. A parallel plate platform was inserted perpendicularly to a coil ring. The ring was the source of PEMF irradiation. Treated cells were placed on the designed platform for irradiation, while at the same time, the control samples were placed in a culture cell hood.

### 3.3. Cytotoxicity Test (MTT Assay)

In order to investigate the possible cytotoxic effect of PEMFs on cells, the MTT colorimetric assay (3-(4,5-dimethylthiazol-2-yl)-2,5-diphenyl-tetrazolium bromide) was performed (Thiazolyl Blue Tetrazolium Bromide M5655, Sigma-Aldrich, Darmstadt, Germany). A multi-well scanning spectrophotometer (enzyme-linked immunosorbent assay reader) was used to quantify cell viability based on the MTT assay. Briefly, the higher the number of viable cells in each well, the higher the optical density value [51]. MCF-7, MDA-MB-231 and FF95 cells, after reaching 70% confluency, were seeded at approximately 10,000 cells/well in 96-well plates. By the end of all treatments, the plate confluency was approximately 80–85%. On the day of the MTT assay, culture medium in each well was replaced with 100 μL of fresh medium. Then, 10 μL of 3-(4,5-dimethylthiazol-2-yl)-2,5-diphenyl-terazolium bromide (MTT) solution [5 mg/mL in phosphate-buffered saline (PBS) (Gibco BRL, Life Technologies, ThermoScientific, Paisley, UK)] was added to each well. Samples were incubated at 37 °C for 2 h. Afterwards, the supernatant was removed from each well, and 100 μL of dimethyl sulfoxide (DMSO) was gradually supplemented and incubated on a shaker for 30 min at room temperature (RT). For background normalization, the optical density was measured at 570 nm and 650 nm, respectively [52]. The percentage viability of the PEMF-treated cells was calculated and compared to that of the untreated control.

### 3.4. Immunocytochemistry for Anti-Biotin, p21^WAF1/Cip1^, and Ki67 Staining

Cells from each cell line were seeded on coverslips, in 100 mm Petri dishes at 50% confluency, to achieve approximately 70–85% confluency by the end of the 5-day period of PEMF treatment. Cell culture medium was replaced on the third day of treatment. Cells were then fixed using 4% Paraformaldehyde (PFA) for 10 min, at 4 °C, and washed with PBS three times. For the Immunocytochemistry (ICC) process, cell membranes were permeabilized using Triton-X 0.3%/PBS for 15 min at RT, followed by the blocking of non-specific binding sites with goat serum (Abcam ab138478, in 1:40) for 1 h at RT. Cells were then incubated with one of the following primary antibodies for 1 h at RT: anti-biotin (Cat.no: ab201341, dilution 1:300, Hyb-8, Abcam, Cambridge, UK), p21^WAF1/Cip1^ (Cat.no: 2947S, dilution 1:400, 12D1, Cell Signaling, Danvers, MA, USA) or Ki67 (Cat.no: ab16667, dilution 1:250, SP-6, Abcam, Cambridge, UK). Signal development was obtained using the Dako REAL EnVision Detection System kit (Cat.no: K5007, Santa Clara, CA, USA) according to the manufacturer’s instructions using 3,3′-Diaminobenzidine (DAB) (brown color). Coverslips were counterstained with hematoxylin and positive cells were counted. Finally, coverslips were sealed and observed under a ZEISS Axiolab5 (Munich, Germany) optical microscope with 20× or 40× objectives [26].

### 3.5. GL13 (SenTraGor^TM^) Staining

For SenTraGor^TM^ staining, as described previously, after the step for blocking of non- specific binding sites, the coverslips were incubated sequentially in 50% and 70% ethanol for 5 min, respectively [26]. After applying GL13 on each coverslip, the samples were incubated at 37 °C for 10 min. At the end of this step, coverslips were washed with 50% ethanol for 30–60 s, with PBS and then Triton-X 0.3%/PBS was applied for 5 min in order to remove any reagent precipitates. Cells were washed again with PBS and anti-biotin antibody (Cat. no: K5007, in dilution 1:300, Hyb-8, ab201341, Abcam, Cambridge, UK) was applied and incubated for 1 h at RT [26]. The mean percentage of GL13-positive cells was estimated from ≥5 high-power fields (Objection 40×) per sample.

### 3.6. Estimation of Cell Proliferation Rate

To test the effect of PEMFs on the proliferation rate, each cell line was seeded accordingly, in order not to exceed 70–85% confluency by the end of the 5 days of PEMF treatment. Cell culture medium was replaced on the third day. To facilitate cell counting, approximately 400,000 cells/Petri dish were seeded for MCF-7 and FF95, while for MDA-MB-231, the seeded number of the cells was 250,000 cells/Petri, since they are more proliferative than MCF-7 and FF95. For each cell line, two different groups of samples were tested, the PEMF-treated cells and the control group. The latter remained in the laminar hood without undergoing PEMF treatment. Following everyday treatment, cells from both groups were counted. Trypan Blue staining and cell counting (Neubauer, Corning, The Netherlands) using a hemocytometer and optical microscope (OLYMPUS IM, Olympus Deutschland GmbH, Hamburg, Germany) was performed every 24 h to obtain the proliferation rate curves [53].

### 3.7. Statistical Analysis

The Wilcoxon signed-ranks test (the non-parametric equivalent of the paired *t*-test) was applied for the statistical analysis of the results (MTT cytotoxicity assay, immunohistochemical stainings). Also, a Two-Way RM (repeated measures) ANOVA test was applied for the evaluation of proliferation rate. *p* < 0.05 was considered statistically significant.

## 4. Conclusions

Although our recommended approach based on PEMF irradiation exhibited obvious advantages, it needs improvement. Regarding the optimization of the whole process, in order to establish a possible therapeutic protocol based on PEMF irradiation, further investigation of the effect of the associated parameters, such as the time of exposure, time the interval between two consecutive exposures, intensity, wave type, other cellular models, etc., should be implemented to ensure the effectiveness on cancer cells.

**Figure 6:**
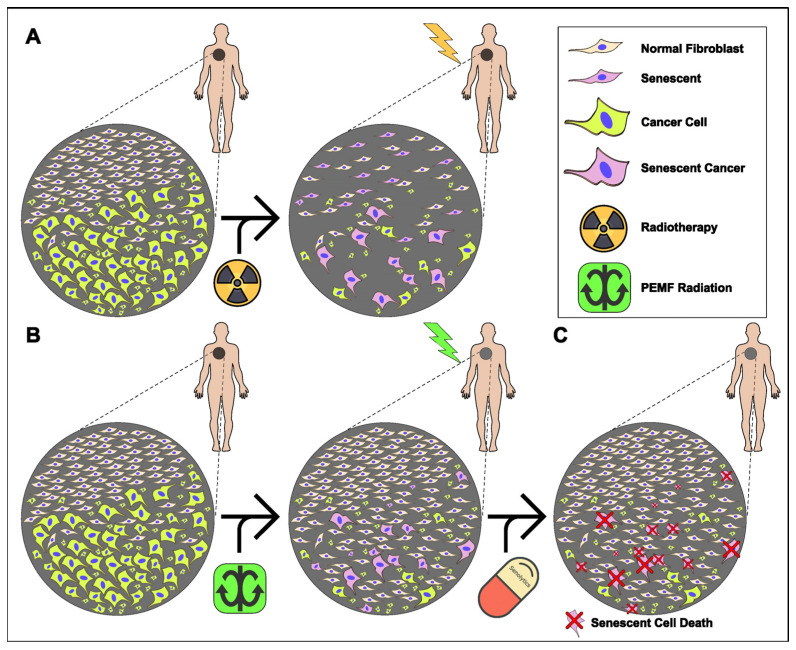
Proposed model for an anti-tumor, combined therapy applying PEMFs and senolytic drugs. (**A**) Traditional radiotherapy induces cell death and/or cellular senesce in both cancer and adjacent normal tissue. (**B**) PEMF irradiation enhances proliferation of normal cells while simultaneously pre-conditioning cancer tissue, through induction of senescence and apoptosis. (**C**) Administration of senolytic drugs eliminates remaining senescent cancer cells following PEMF treatment.

In conclusion, PEMF irradiation is a promising pre-conditioning, non-invasive strategy for tumor elimination, limiting the side effects of traditional radiotherapy (Figure 6A), as revealed by our findings. PEMF-treated cancer cells not only displayed cell death but also senescence. In contrast, normal fibroblasts were not affected by the possible harmful effects of PEMFs, but increased their cellular viability (Figure 3C). These findings suggested that PEMFs reduced the viability of cancer cells without having side effects on the adjacent normal tissue (Figure 6B), an outcome that is observed during traditional radiation therapy. Finally, a two-step treatment consisting of PEMF irradiation followed by the targeted administration of a senolytic drug could alter the route of cancer therapeutics (Figure 6C).

## Figures and Tables

**Table 1:** Settings of the different tested PEMF programs that were applied in the experimental procedure.

	Pre-Set Program C4	Manual Program 1	Manual Program 2	Manual Program 3
**Frequency**	8–31 Hz	8 Hz	8 Hz	8 Hz
**Wave Type**	Sinus-Rechteck	Full square	Full square	Full square
**Intensity**	0.0033–0.0055 T	0.0088 T	0.0044 T	0.011 T
**Duration**	30 min (2 times)/5 days	1 h (2 times)/5 days	30 min (2 times)/5 days	30 min (2 times)/5 days

## Data Availability

Data is contained within the article.
